# A Case Report on the Atypical Presentation of Hypertrophic Cardiomyopathy (HOCM) in a 19-Year-Old Female

**DOI:** 10.7759/cureus.33136

**Published:** 2022-12-30

**Authors:** Ahmed Alhazmi, Shahad B Almatrafi, Rahaf A Abdulwahab, Asma Alzahrani, Ghufran Sindi

**Affiliations:** 1 Cardiology, King Abdullah Medical City, Makkah, SAU; 2 College of Medicine, Umm Al-Qura University, Al-Abdia Main Campus, Makkah, SAU; 3 Medicine and Surgery, Umm Al-Qura University, Makkah, SAU

**Keywords:** saudi arabia, case report, hypertrophic cardiomyopathy, cardiology, hocm

## Abstract

Atypical hypertrophic cardiomyopathy (HOCM) is a relatively rare genetic disorder that can affect the left ventricular system. HOCM can lead to various cardiac issues such as sudden cardiac death (SCD). We report a case of a 19-year-old female who was referred to a cardiology clinic after presenting with bi-ventricular hypertrophy on an echocardiogram (ECHO). Results from screening tests for infiltrative diseases and an iron panel came negative. The patient was asymptomatic, with no functional limitations and no family history of any cardiac disease or sudden death. In conclusion, HOCM can present with an atypical pattern, such as biventricular hypertrophy, and has been linked to SCD; therefore, it is important to be aware of this condition and take the necessary precautions to prevent it.

## Introduction

Atypical hypertrophic cardiomyopathy (HOCM) is a relatively rare genetic disorder that can affect the left ventricular system. HOCM can lead to various cardiac issues such as sudden cardiac death (SCD) [1,2]. The recognition of HOCM is a vital part of the treatment and prevention of this condition, it can also trigger the initiation of genetic surveillance and clinical procedures for family members [3].

HOCM symptoms are described as lightheadedness, presyncope, syncope, and sudden death. Also, exercise-induced dyspnea can be caused by a non-compliant left ventricle (LV) with a low end-diastolic volume that limits cardiac output [4]. Increased awareness of HOCM will further help improve the quality of life for many patients and increase the likelihood of diagnosis. Furthermore, this awareness will lead to lower mortality in adult patients with this disease in the general population [5]. In the current report, we discuss an atypical presentation of HOCM in a young healthy 19-year-old girl who presented to our hospital in Makkah, Saudi Arabia.

## Case presentation

A 19-year-old female, with no previous medical or surgical history, was referred to our cardiology clinic after presenting with bi-ventricular hypertrophy on an echocardiogram (ECHO) during her regular visit for anemia monitoring in the hematology department. The patient had no previous complaints and was doing regular daily activities. She denied a history of dyspnea, palpitation, syncope, or numbness. Family history was insignificant, except for two siblings with thalassemia. Otherwise, no family history of sudden cardiac death or cardiac disease was reported. On physical examination, she was vitally stable, appeared well, and was conscious, oriented, and not in distress. Cardiac examination yielded normal S1 and S2 without an audible murmur. Lung examination revealed equal bilateral air entry with no added sounds. No skin lesions or visible deformities were detected. Results from screening tests for infiltrative diseases and iron panel were negative for Fabry disease, hemochromatosis, sarcoidosis, and amyloidosis. The renal function test was normal.

**Figure 1:**
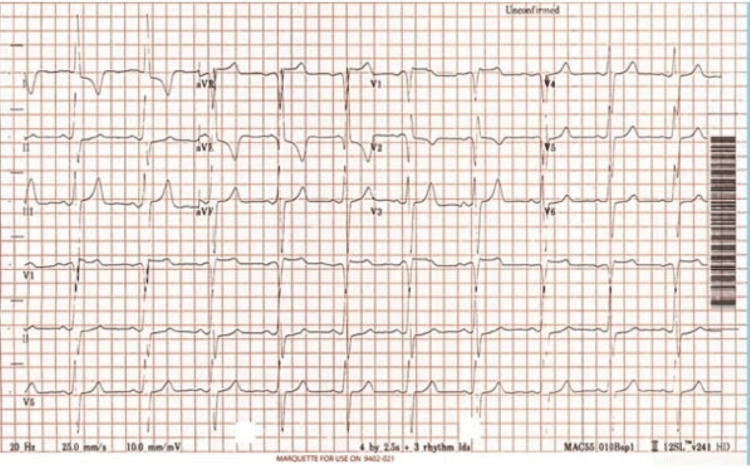
Electrocardiography (ECG) of the patient presenting Cornell voltage

**Figure 2:**
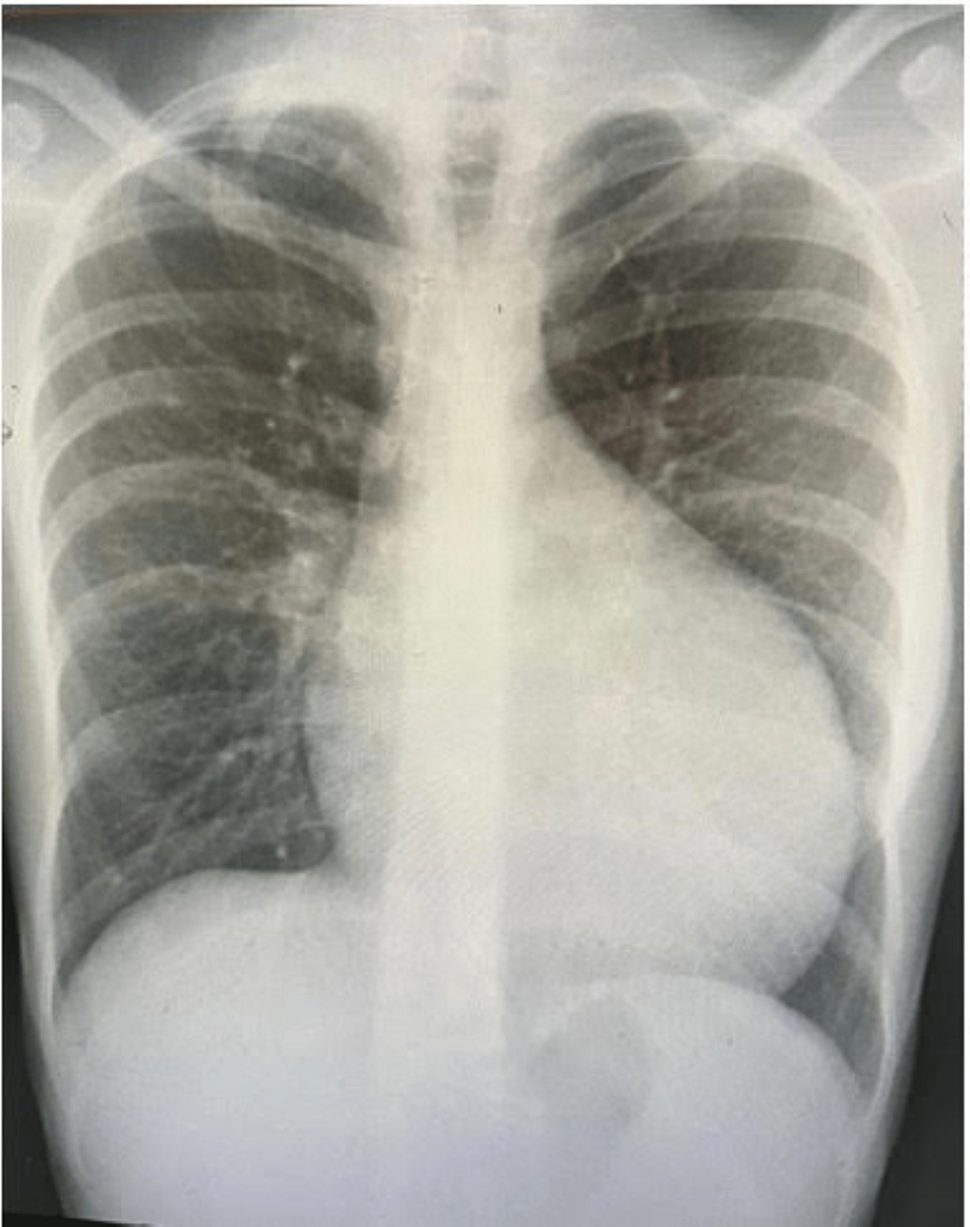
Posterior-anterior (PA) view of the chest X-ray showed an enlargement of the cardiac silhouette

**Figure 3:**
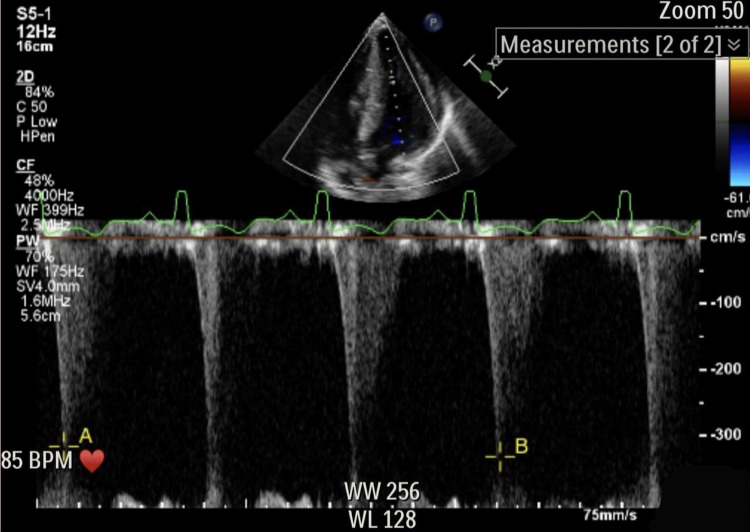
A left ventricle mid-cavity gradient of 36 mmHg through an echocardiogram (ECHO) using a pulse wave Doppler

Electrocardiography (ECG) of the patient revealed Cornell voltage, which is an S wave in lead V3 and R wave in aVL more than 30, as shown in Figure 1. On imaging, a chest X-ray showed an enlargement of the cardiac silhouette (Figure 2). In addition, an echocardiogram (ECHO) five-chamber view using a pulse wave Doppler was done for the patient. It revealed severe concentric left ventricular hypertrophy with an intramyocardial gradient of 36 mmHg, severe right ventricular hypertrophy with normal biventricular systolic functions, and an estimated ejection fraction (EF) of the left ventricle of more than 55% (Figure 3).

**Figure 4:**
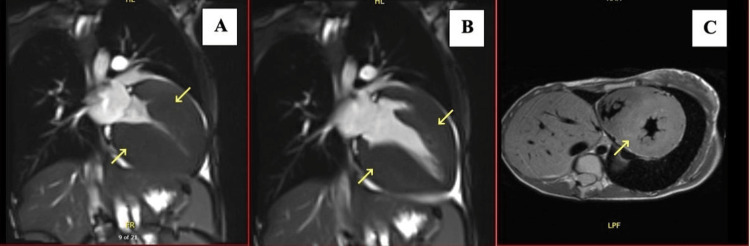
Cardiac magnetic resonance imaging (CMRI) Two-chamber view showing the left ventricle (LV) during systole through a balanced steady-state free-precession (bSSFP) (4A); two-chamber view showing LV during diastole through a bSSFP (4B); T2-weighted short axis view showing both ventricles (4C)

For further evaluation, we ordered cardiac magnetic resonance imaging (CMRI) with contrast, which revealed severe right ventricular hypertrophy (EF 59%). In addition, severe concentric left ventricular hypertrophy with a maximum thickness of 39 at the septum and an inferior wall was detected; there was no systolic anterior motion of the mitral valve, left ventricular EF was 71%, and left ventricular short-axis image obtained by T1-weighted CMRI showed mid-wall delayed enhancement of the left ventricle suggestive of hypertrophic cardiomyopathy (Figure 4).

Genetic testing for the molecular genetic analysis of known genes associated with HOCM was sent; nevertheless, it currently remains unclear whether the detected heterozygous variant of uncertain significance (VUS) in gene MYH7 might be responsible for the clinical phenotype of our patient (Table 1).

**Table 1:** Geneticizing for the molecular genetic analysis

Gene isoforms	Phenotype MIM number (mode of inheritance)	Variant	Zygosity	MAF gnomAD (%)	Classification
MYH7 (NM_000257.4)	192600(AD) 613426 (AD)	c.4520-3C>T P.? Chr14:23886204	het	0.0055	Uncertain significance

The patient is still asymptomatic. We offered an implantable cardioverter-defibrillator (ICD) insertion, as her left ventricle thickness was more than 30 mm but she was reluctant. Due to the eligibility policy, the family was advised to be screened in a primary hospital.

## Discussion

HOCM is a major cause of SCD. Relative myocardial ischemia is thought to lead to variable degrees of myocardial ischemia, cell death, and fibrosis over time [6]. HOCM is estimated as 1:500 in young adults and is augmented in families; this estimate is based on a demographic survey of people aged 23 to 35 from the general population of four urban centers [7]. Hypertrophy of the ventricular myocardium occurs in response to both physiological and pathological stimuli such as hypertension or aortic stenosis [7]. The identification of a sarcomere mutation in a large family with HOCM, sudden death, and heart failure in the 1990s provided a description of the underlying genetic etiology of HOCM. The HOCM phenotype can be caused by mutations in any of the genes that produce cardiac contractile proteins. Despite the fact that echocardiography is frequently used for HOCM screening, CMR is more sensitive for detecting atypical locations of hypertrophy and apical HOCM. Since a higher LV mass is linked to worse outcomes, it is crucial that it is used as the most accurate way of determining LV mass. CMRI, like ECHO, can quantify the LV outflow gradient using velocity-encoded imaging and assess mitral regurgitation. It can also detect systolic anterior motion of the mitral valve. CMR is an effective tool for assessing patients who have or are at risk of developing heart failure and has an impact, increasing clinical management, research, and diagnosis [8].

The earliest sign of diastolic dysfunction in patients with thalassemia major and normal systolic function who have iron overload is a left ventricular relaxation impairment that manifests as a prolonged isovolumic relaxation time [9]. Stage I of the disease is defined by the onset of left ventricular hypertrophy (LVH) with or without left ventricular outflow tract (LVOT) obstruction, hyperdynamic ventricular function, and mild symptoms like decreased exercise tolerance or intermittent chest pain. Adverse myocardial remodeling with increasing myocardial hypertrophy and fibrotic changes takes place during stage II of the disease. The irreversible "end stage" of the disease, Stage III carries a high risk of morbidity and mortality. This stage is characterized by severe LV fibrosis, progression to LV dilatation, atrial dilatation, and systolic and diastolic dysfunction linked to hemodynamic decompensation, complications from heart failure, heart transplantation, and/or death. New imaging modalities and molecular diagnostics have improved patient management. A significant decrease in HCM mortality has been achieved through the application of current standard therapeutic measures such as prohibition, competitive sports participation, and implanting cardioverter-defibrillators when necessary in addition to therapies for symptomatic heart failure or cardiac transplantation (Table 2).

**Table 2:** Differential diagnosis of left ventricular hypertrophy (LVH) CMR: cardiac magnetic resonance imaging; TTE: transthoracic echocardiogram; ECG: echocardiogram; HOCM: hypertrophic cardiomyopathy; GLA: alpha-galactosidase gene

	DISEASE	GENE	PROTEIN	PHENOTYPE	WORK-UP
1	Anderson-Fabry disease (AFD)	GLA	α-galactosidase A	Multisystem, involving skin, kidney, and peripheral nerves, X-linked inheritance	Molecular genetics, histopathology, CMR, enzyme assay, and other organ involvement
2	HOCM (athlete’s heart)	-	-	Concentric hypertrophy, enlarged ventricular cavity, no diastolic or systolic dysfunction, reversible	Classical features on TTE and ECG, no fibrosis on CMR, reversible after deconditioning
3	Loading conditions (pressure or volume overload), Complication of thalassemia	-	-	Structural heart disease	Transthoracic echocardiography

Since there is no hypothesized treatment for HCM at the moment, current management focuses on the early detection of at-risk asymptomatic patients using clinical cascade screening of family members and molecular diagnostics, optimum sudden death risk stratification, and timely initiation of preventative therapies to prevent disease progression to the irreversible adverse myocardial remodeling stage [10]. Anderson-Fabry disease (AFD) is a rare X-linked inborn error of glycosphingolipid catabolism caused by mutations in the alpha-galactosidase A gene (GLA) at Xq22. AFD is caused by abnormal glycosphingolipid metabolism caused by lysosomal-galactosidase A activity. AFD is a multiorgan systemic disease, and various studies have shown that endothelial damage is the primary pathological action. Fabry and Anderson described this pathology for the first time in 1898. The latter was a representation of angiokeratoma corporis diffusum in males. AFD is caused by a lack of galactosidase A, which causes an accumulation of Gb3, its metabolite globotriaosylsphingosine (Lyso-Gb-3), and its precursor metabolite Gal-Gal-Cer in the lysosomes [11]. Currently, the two clinically available therapeutic modalities for the treatment of AFD are enzyme replacement therapy (ERT) and chaperone therapy with other alternatives such as substrate reduction therapy, mRNA-based therapy, and gene therapy in development.

## Conclusions

The condition known as HOCM is a genetic disorder that affects the left ventricular system. It is also known to cause sudden cardiac death. Being aware of this condition and taking the necessary precautions to prevent it are two of the most important factors that people can consider. CMR is a good tool that can be used for the better assessment of HOCM and can be used in some patients with an atypical presentation to exclude infiltrative disease for which the diagnosis is still not confirmed by ECHO.

## References

[1] Maron BJ, Maron MS (2013). Hypertrophic cardiomyopathy. Lancet.

[2] Maron BJ, Ommen SR, Semsarian C, Spirito P, Olivotto I, Maron MS (2014). Hypertrophic cardiomyopathy: present and future, with translation into contemporary cardiovascular medicine. J Am Coll Cardiol.

[3] Maron BJ, Gardin JM, Flack JM, Gidding SS, Kurosaki TT, Bild DE (1995). Prevalence of hypertrophic cardiomyopathy in a general population of young adults. Echocardiographic analysis of 4111 subjects in the CARDIA study. Circulation.

[4] Frenneaux MP, Counihan PJ, Caforio AL, Chikamori T, McKenna WJ (1990). Abnormal blood pressure response during exercise in hypertrophic cardiomyopathy. Circulation.

[5] Basu J, Malhotra A, Papadakis M (2020). Exercise and hypertrophic cardiomyopathy: two incompatible entities?. Clin Cardiol.

[6] Gastl M, Gruner C, Labucay K (2020). Cardiovascular magnetic resonance T2* mapping for the assessment of cardiovascular events in hypertrophic cardiomyopathy. Open Heart.

[7] McNally EM, Barefield DY, Puckelwartz MJ (2015). The genetic landscape of cardiomyopathy and its role in heart failure. Cell Metab.

[8] Patel AR, Kramer CM (2017). Role of cardiac magnetic resonance in the diagnosis and prognosis of nonischemic cardiomyopathy. JACC Cardiovasc Imaging.

[9] Gharzuddine WS, Kazma HK, Nuwayhid IA, Bitar FF, Koussa SF, Moukarbel GV, Taher AT (2002). Doppler characterization of left ventricular diastolic function in beta-thalassaemia major. Evidence for an early stage of impaired relaxation. Eur J Echocardiogr.

[10] Wolf CM (2019). Hypertrophic cardiomyopathy: genetics and clinical perspectives. Cardiovasc Diagn Ther.

[11] Tuttolomondo A, Simonetta I, Riolo R, Todaro F, Di Chiara T, Miceli S, Pinto A (2021). Pathogenesis and molecular mechanisms of Anderson-Fabry disease and possible new molecular addressed therapeutic strategies. Int J Mol Sci.

